# Supplementary material for: Sacsin Deletion Induces Aggregation of Glial Intermediate Filaments
Source: Cells. 2022 Jan 16;11(2):299. doi: 10.3390/cells11020299 (PMC8773934; doi:10.3390/cells11020299)
Supplement: Supplementary file 1 [file cells-11-00299-s001.zip › cells-1539182-supplementary.pdf]

Supplementary material:

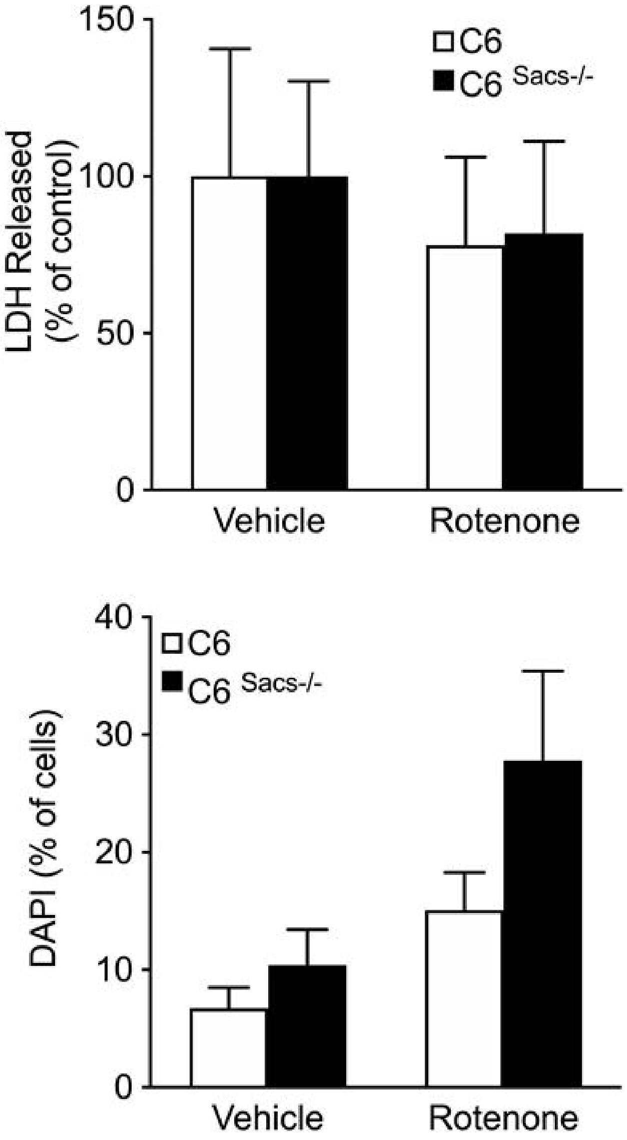

Figure S1. Toxicity of short treatment of rotenone in C6 strains. A, Released LDH was determined after incubation of C6 strains with rotenone 5 μM for 4 h, and normalized versus total LDH and control values. No significant changes in LDH released were registered. B, Incubation with rotenone for 4 h increases slightly the percentage of DAPI-positive cells in both C6 cell strains, the differences not achieving statistical significance. Graphs show the average of 3 independent experiments (mean ± SEM), data were analyzed by means of two-way ANOVA, followed by a Tukey post-hoc test. Significance threshold was $p<0.05$.

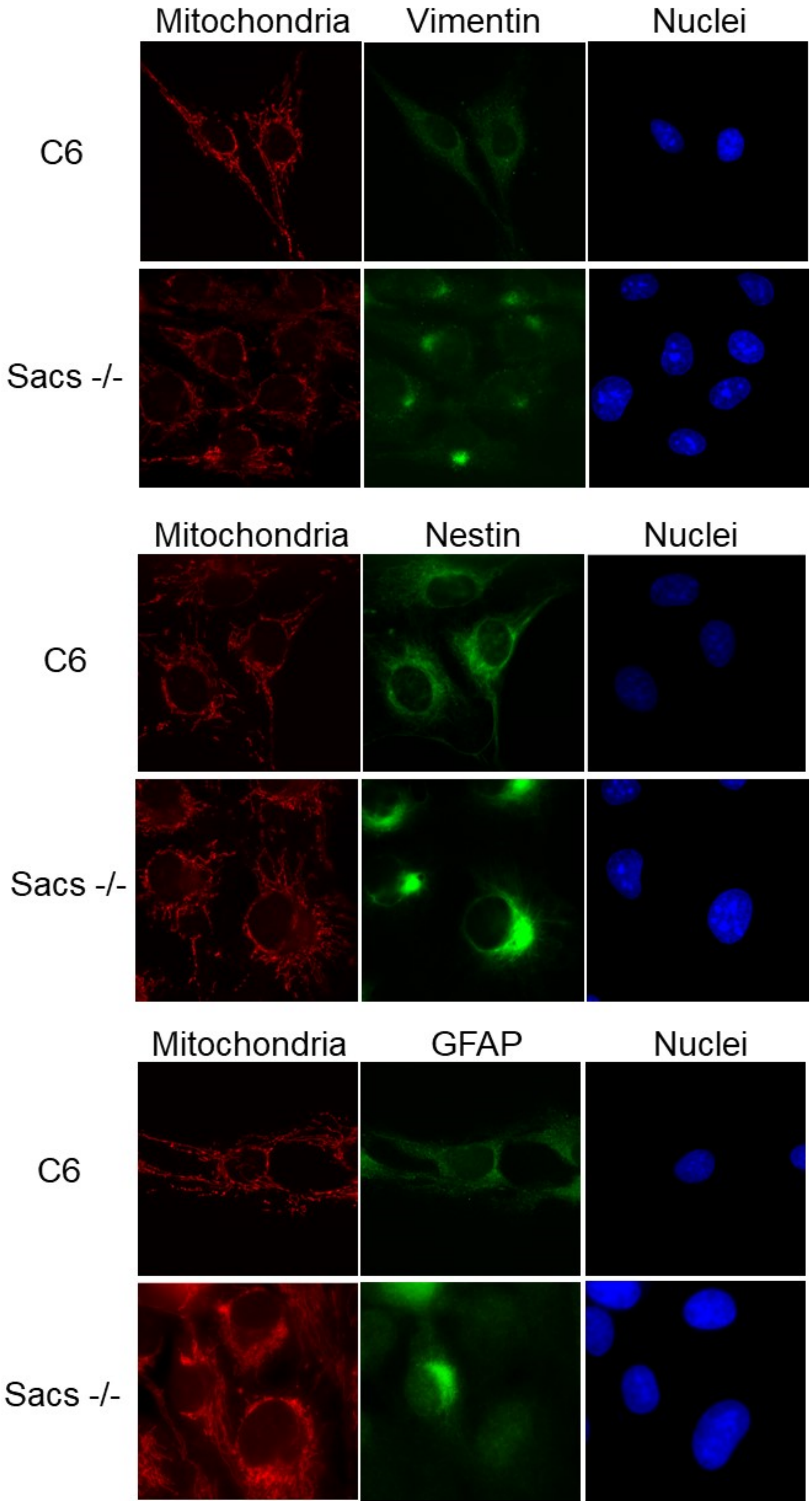

Figure S2. Separation of channels corresponding to Figure 3A. Representative immunocytochemistry images showing the distribution of the glial intermediate filaments vimentin, nestin and GFAP (green), mitochondria (Mitotracker, red) and nuclei (Hoechst, blue) in C6 and C6Sacs-/- cells. Vimentin, nestin and GFAP accumulate in the juxtanuclear area in C6Sacs-/- cells.

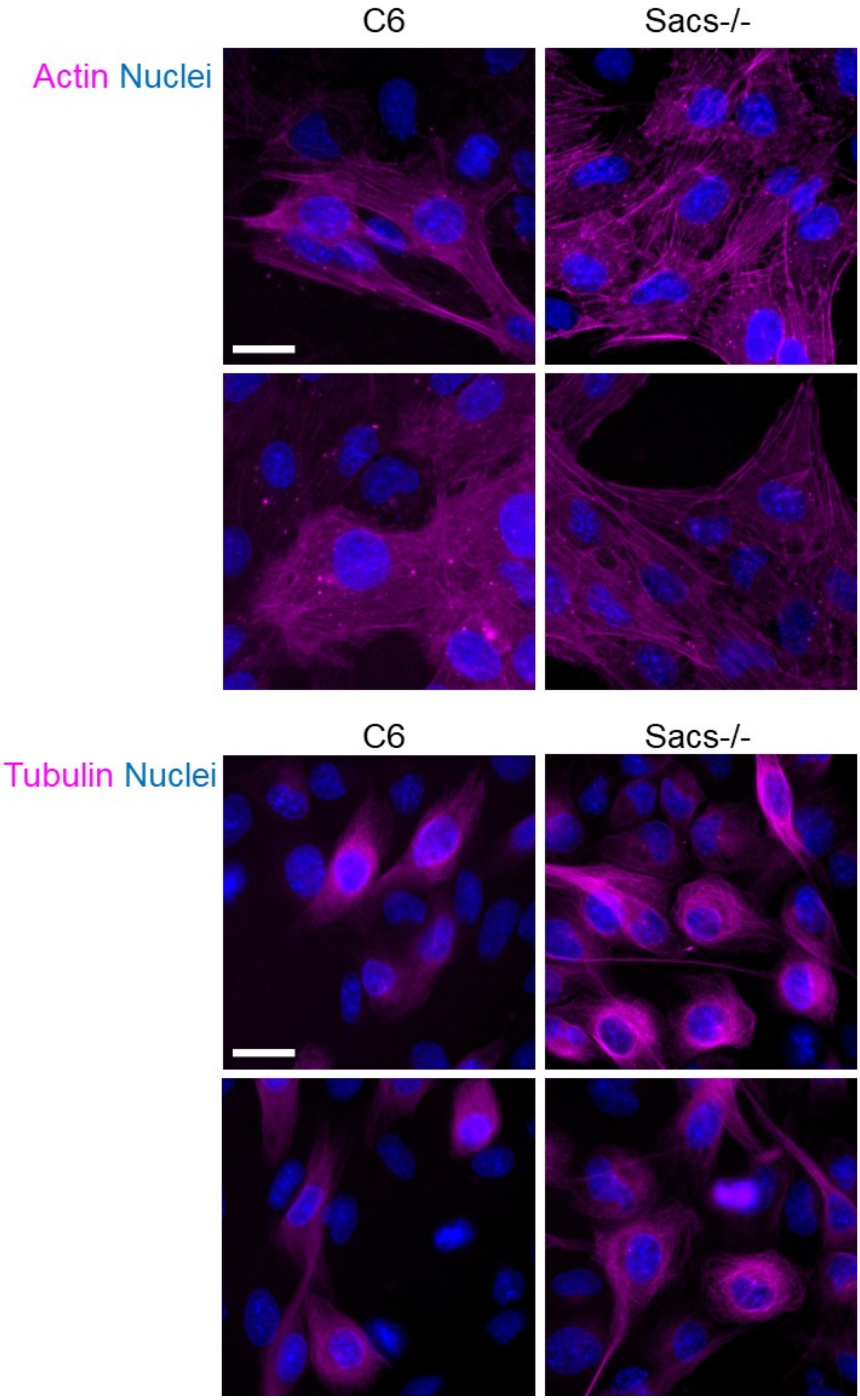

Figure S3. Sacsin knockout does not produce gross changes in actin and microtubule networks. Representative immunocytochemistry images showing the distribution of actin microfilaments or microtubules (magenta) and nuclei (Hoechst, blue) in C6 and C6Sacs-/- cells. Live cells were stained with sirActin kit (Spirochrome) and Tubulin tracker deep red (Invitrogen) following manufacturer´s instructions. Scale bar, 20 μM.

Figure 1A

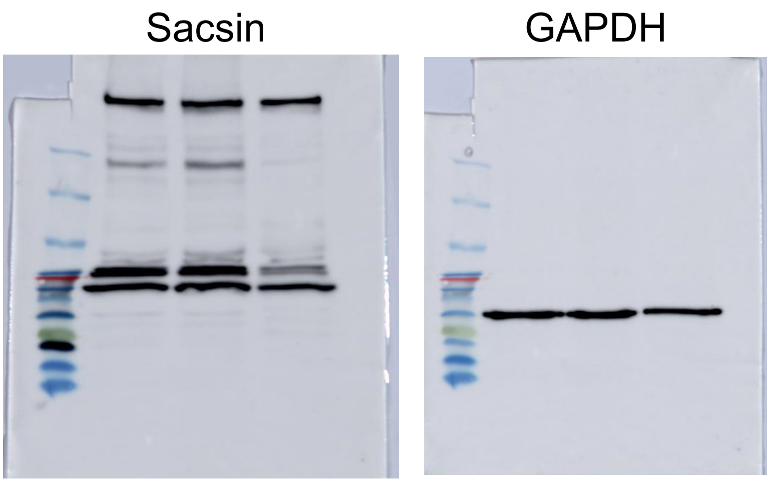

Figure 1B

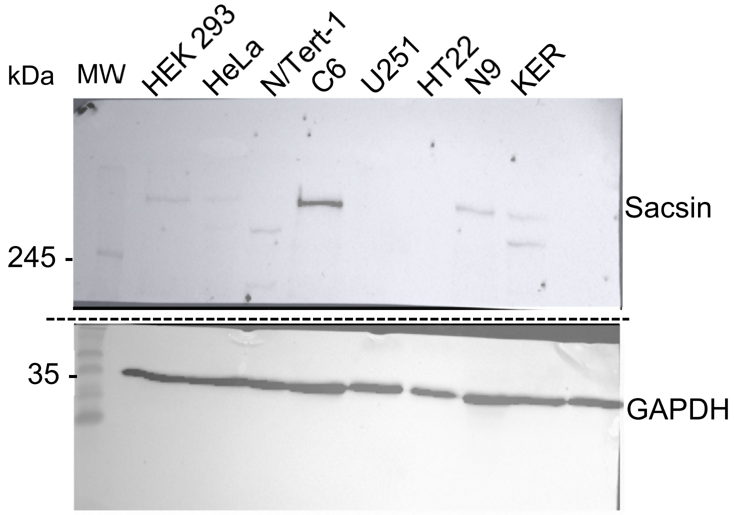

Figure 1E

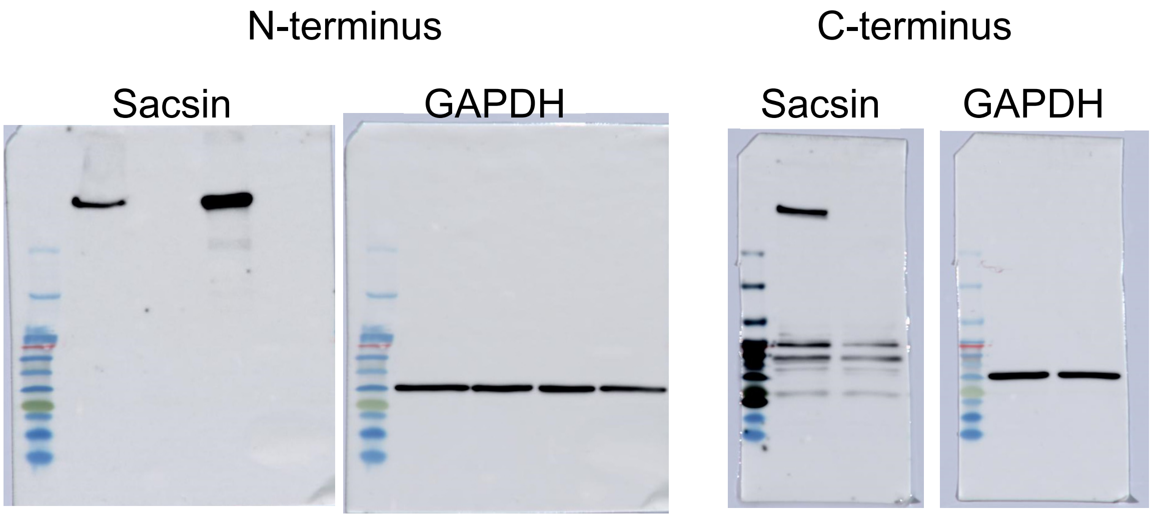

Figure S4. Full membranes and molecular weight markers for western blots in Figure 1.

Figure 3D

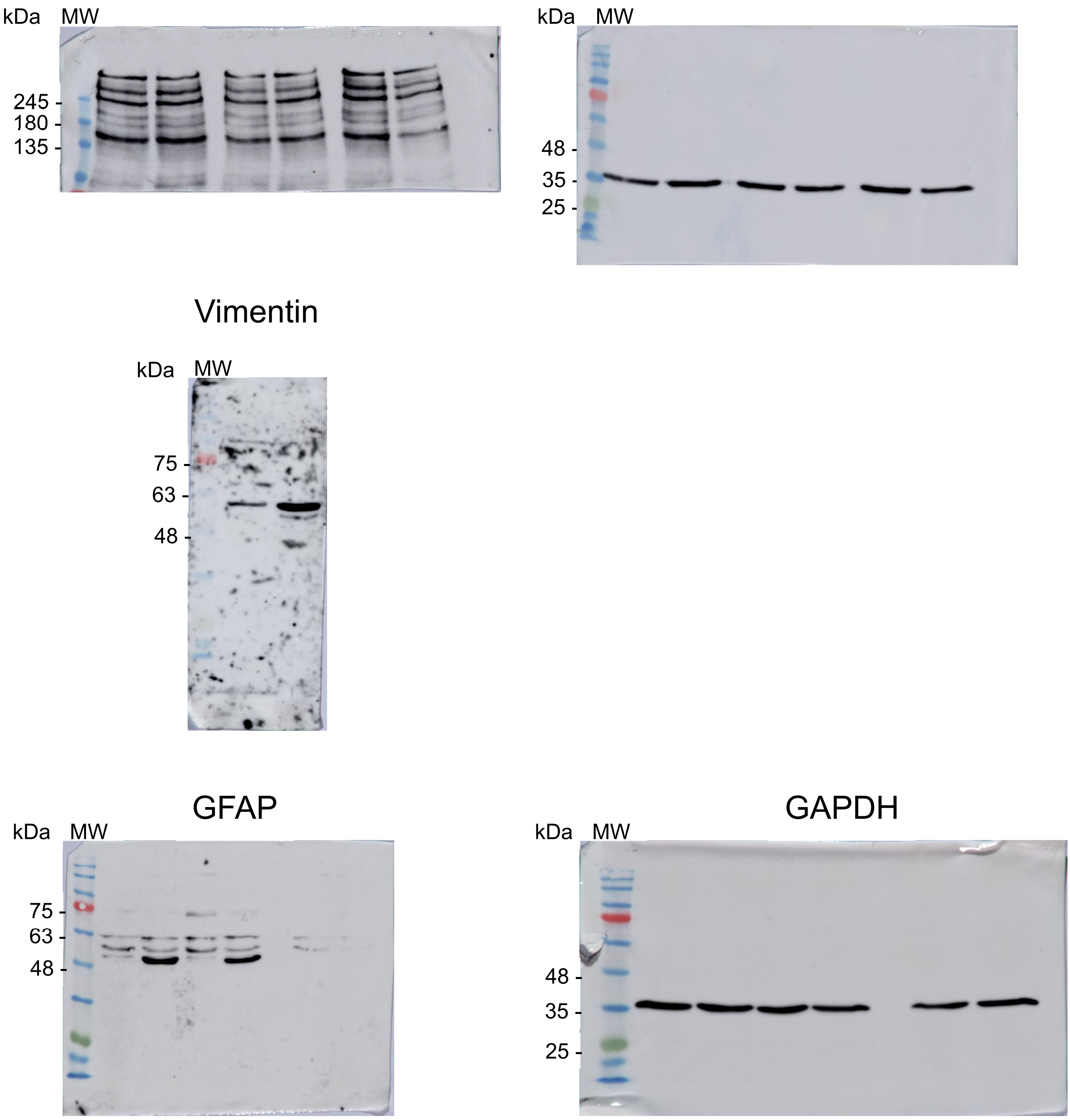

Figure S5. Full membranes and molecular weight markers for western blots in Figure 3.

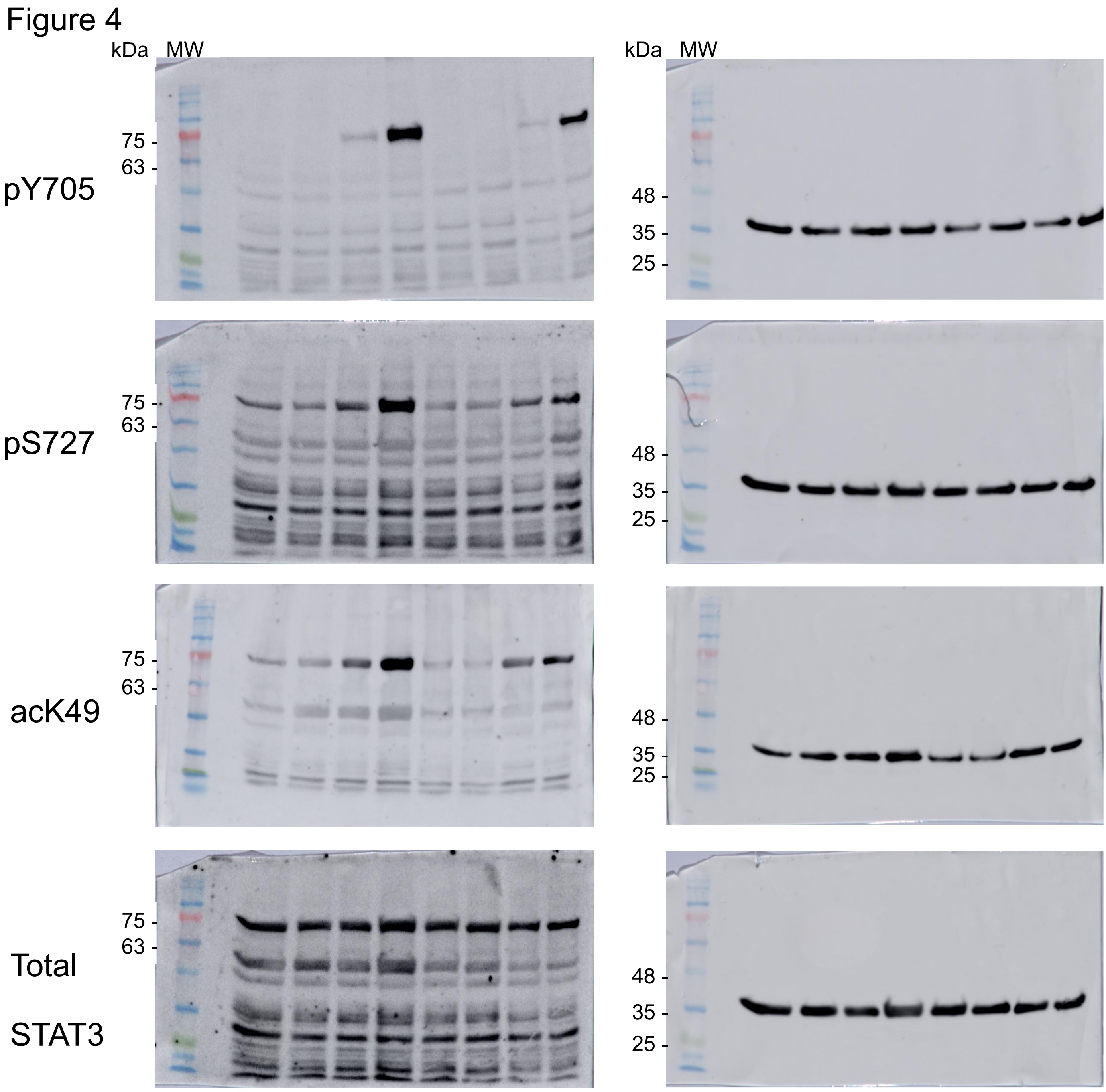

Figure S6. Full membranes and molecular weight markers for western blots in Figure 4.
